# Long non-coding RNA ABHD11-AS1 promotes colorectal cancer development through regulation of miR-133a/SOX4 axis

**DOI:** 10.1042/BSR20181386

**Published:** 2018-12-14

**Authors:** Xiaoyan Lei, Longchao Li, Xiaoyi Duan

**Affiliations:** 1 Radiology Department, First Affiliated Hospital of Xi’an Jiaotong University, Xi’an city, Shaanxi Province, P.R. China; 2 Department of MRI, Shaanxi Provincial People’s Hospital, Xi’an city, Shaanxi Province, P.R. China

**Keywords:** colorectal cancer, large intervening non-coding RNA, microRNA, tumorigenesis

## Abstract

Recently, lncRNA has been verified to regulate the development and progression of tumor. LncRNA ABHD11-AS1 has been proven to serve as an oncogene in several cancers. However, the role of ABHD11-AS1 in colorectal cancer remains totally unknown. In the present study, qRT-PCR assay revealed that ABHD11-AS1 expression was markedly higher in colorectal cancer tissues and cell lines. In addition, patients who displayed overexpression of ABHD11-AS1 showed a significantly poorer progression free survival (PFS) and overall survival (OS) by Kaplan–Meier analysis. Loss-of-function experiments suggested that silencing of ABHD11-AS1 expression could significantly reduce the proliferation, colony formation, migration and invasion of colorectal cancer cells, and increase cell apoptosis. Moreover, bioinformatics analysis, biotin pull-down assay, luciferase reporter assay, and RIP assay disclosed that ABHD11-AS1 straightly interacted with miR-133a. We also found that SOX4 was a downstream target of miR-133a and ABHD11-AS1 subsequently exerted its biological effects via modulating the expression of SOX4 in colorectal cancer cells. Collectively, these findings manifested that the ABHD11-AS1/miR-133a/SOX4 axis may be a cogitable and promising therapeutic target for colorectal cancer.

## Introduction

Colorectal cancer (CRC) is the most common digestive system malignancy worldwide and caused by various factors such as environmental, genetic, and epigenetic ones [1]. Although surgery, chemotherapy, radiotherapy, targeted therapy, and immunotherapy have reduced the relapse and improved the survival of CRC patients, the 5-year survival rate of this cancer is still poor [1–3]. Therefore, it is urgent to identify effective biomarkers and therapeutic target by understanding the mechanism of CRC tumorigenesis and progression. Recently, increasing evidences have showed that long non-coding RNA (lncRNAs, more than 200 nt), such as HOTAIR, H19, and TUG1, plays an important role as oncogene or suppressor in the tumorigenesis of CRC [4–6]. However, the functions of most lncRNAs in CRC remain unclear.

LncRNA ABHD11 antisense RNA 1 (ABHD11-AS1), locating at the human chromosome 7 q11.23, has been identified as one oncogene in various cancer [7–10]. For instance, Liu et al. demonstrated that lncRNA ABHD11-AS1 stimulates cell proliferation and invasion of endometrial carcinoma [7]. Moreover, lncRNA ABHD11-AS1 promotes the tumorigenesis and progression of epithelial ovarian cancer through targeted regulation of RhoC [8]. In addition, ABHD11-AS1 is up-regulated and induces cell proliferation and cell migration in bladder cancer and gastric cancer [9,10]. However, the role and mechanism of ABHD11-AS1 in the tumorigenesis of CRC remain to be uncovered. Besides, it has been reported that miR-133a acts as tumor suppressor to inhibit the tumorigenesis of CRC [11,12]. Otherwise, miR-133a suppresses the migration and invasion of esophageal cancer cells by targeting the SOX4 [13]. Additionally, it has been reported that SOX4 promotes proliferation, migration, and invasion of CRC cells [14–17]. However, whether miR-133a could inhibit progression of CRC cells via targeting SOX4 remains unknown.

In the present study, we for the first time showed that ABHD11-AS1 was significantly up-regulated in CRC tissues and cell lines. Additionally, we found that high expression of ABHD11-AS1 positively shorten the overall survival (OS) and progression-free survival (PFS) of the patients with CRC. *In vitro*, we found that ABHD11-AS1 promoted proliferation and migration, and inhibited the apoptosis of CRC cells. Mechanistically, we manifested that ABHD11-AS1 sponged miR-133a to inhibit cell proliferation and migration of CRC by regulating SOX4 expression. In short, our data for the first time established the crucial functions of ABHD11-AS1/miR-133a/SOX4 axis in CRC, which may provide a new way for the therapy of CRC.

## Materials and methods

### Patients and tissue samples

Paired CRC tissues and adjacent normal tissues were collected between Jan 2012 and May 2013 from 132 patients who underwent surgery at Shaanxi Province People’s Hospital. All biopsy specimens were directly frozen in liquid nitrogen and stored at −80°C. All patients received neither chemotherapy nor radiotherapy before surgical resection. All patients signed the informed consents for the utilization of tissue samples in research. The present study was carried out under the supervision of the Ethics Committee of Shaanxi Province People’s Hospital.

### Cell culture and cell transfection

All the CRC cell lines (SW480, HT-29, LoVo, HCT-116, HCT-8, SW620, Caco-2) and normal human colonic epithelial cell (HcoEpiC) were furnished from the American type culture collection (ATCC) (Maryland, USA) and cultured in RPMI 1640 (Sigma) medium supplemented with 10% fetal bovine serum (Gibco), 1% penicillin/streptomycin (HyClone). All the cell were cultivated in humidified chamber at 37°C with 5% CO2.

SiRNA were obtained from Sigma-Aldrich (Sigma). miR-133a mimics, miR-133a inhibitor as well as the negative control were all obtained from GenePharma (Shanghai, China). Cells were transfected with Lipofectamine 3000 (Life Technologies) according to the manufacturer’s guide. The ABHD11-AS1 siRNA sequences were as follows: forward, 5′-GCUACGAGAUCAUGAGCCA-3′ and reverse, 5′-UGGCUCAUGAUCUCGUAGC-3′.

### Quantitative real-time PCR (qRT-PCR) assay

TRIzol reagent (Invitrogen) was used to purify total RNA and cDNA was synthesized using SuperScript First-Strand Synthesis System (Invitrogen) according to the manufacturer’s guide. Quantitative RT-PCR was carried out using LightCycler480 II Sequence Detection System (Roche) to measure the relative expression of target gene. GAPDH or U6 was used as the internal normalizer for target genes or miRNA, respectively. All the primers were designed and synthesized by Shanghai Shenggong Biotechnology (Shanghai). Relative expression of target genes and miRNA expression was calculated using 2^−ΔΔCt^ method.

### Cell proliferation assay

Luminescent CellTiter-Glo assay (Promega) was used to evaluate the cell viability by detecting the cellular ATP levels according to the manufacturer’s guide. Briefly, CRC cells were seeded into 96-well plates at a density of 1000–2000 cells/well. As the indicated time after transfection, CellTiter-Glo solution was added to each well, and then the luminescent signal was read using a Microplate Reader (EnVision).

### Cell colony formation assay

For clonogenic assay, 200 cells per well of CRC cells were plated in six-well plates and incubated for 2 weeks until visible colonies formed (cell number more than 50). Colonies were washed, fixed with 4% formaldehyde, stained with 0.1% crystal violet, and counted.

### Cell apoptosis detection assay

For cell apoptosis, active caspase 3 human ELISA kit (Invitrogen) was used to detect the cell apoptosis according to the manufacture’s guide. The procedure was the same to the previous description [18].

### Migration and invasion assay

BD 24-well, 8-μm pore size transwell chamber (Costar) without or with Matrigel (20 μg per well; BD) was used to detect the migration and invasion according to the manufacturer’s guide. The procedure was the same to the previous description [19].

### Pull down assay with biotinylated lncRNA ABHD11-AS1 DNA probe

Briefly, the biotin-labeled ABHD11-AS1 DNA probe was designed (Genechem), dissolved in binding and washing buffer and mixed with M-280 streptavidin magnetic beads (Invitrogen) to generate probe-coated beads based on the manufacturer’s guide. Cell lysates were mixed with the probe-coated beads. The RNAs combined to probe-coated beads were washed off and purified using qRT-PCR analysis. The ABHD11-AS1 pull-down probe sequence was 5′-Bio-GCAAGTCTGCTAGGACAGGTCCAG-3′; and random pull-down probe sequence used as negative control was 5′-Bio-TGCATCCAAGCCGATTGCGGTAACG-3′.

### Reporter vectors and dual-luciferase reporter assay

The putative miR-133a target binding sequence in ABHD11-AS1 and its mutant of the binding sites was amplified by PCR and the PCR products were cloned into pMirReporter plasmid (Promega, Madison, USA) to form the ABHD11-AS1-wild-type (WT-ABHD11-AS1) vector and ABHD11-AS1-mutated-type (MUT-ABHD11-AS1). Similarly, the SOX4-wild-type (WT-SOX4) and SOX4-mutated-type (MUT-SOX4) reporter vectors were constructed. Subsequently, mutated or wild-type pMirReporter luciferase vector and miR-133a mimic or NC-mimic were cotransfected into HEK-293 cells in 96-well plates for 24 h with Lipofectamine 3000 (Invitrogen). The luciferase activity was determined by the luciferase reporter assay system (Promega) according to the manufacture’s instruction.

### RNA immunoprecipitation (RIP) assay

The Magna RIP RNA-binding protein immunoprecipitation kit (Sigma) was used to perform RIP assay according to the manufacturer’s protocol. Cells were lysed using RIP lysis buffer. Subsequently, whole cell lysate was incubated with RIP immunoprecipitation buffer containing magnetic beads conjugated with human anti-Argonaute2 (Ago2) antibody (Sigma), and negative control normal mouse IgG (Sigma). Immunoprecipitated RNA was obtained and then the expression levels of ABHD11-AS1 and miR-133a, which presented in the precipitates, was subjected to qRT-PCR analysis.

### Western blot analysis

Cells were collected and lysed with RIPA lysis buffer added with proteinase inhibitors cocktail (Roche). Lysate proteins were separated by a 10% sodium-dodecyl sulfate polyacrylamide gel electrophoresis (SDS-PAGE) and transferred onto PVDF membrane. After blocking with 5% BSA buffer, the blots were incubated with primary antibodies overnight at 4 °C. The primary antibodies used included β-actin antibody (Rabbit, Sigma) and SOX4 antibody (Rabbit, Cell Signaling Technology). Following incubation with appropriate secondary antibody, the bands were scanned using LI-COR-Odyssey scanner (LI-COR). Signal intensity of bands were further analyzed by Image J software.

### Statistical analysis

All data were presented as mean ± SD and all the statistical analysis were processed by GraphPad Prism 5.0. Student’s *t*-test or one-way analysis of variance (ANOVA) were used to analyze the difference between groups for expression of target gene, cell proliferation, colony formation, apoptosis, migration, and invasion. Kaplan–Meier method and the log-rank test was used to evaluate PFS or OS (*P < 0.05, **P < 0.01, ***P < 0.01).

## Results

### ABHD11-AS1 was strongly overexpressed in CRC tissues and cell lines and was significantly associated with poor prognosis of CRC patients

**Figure 1:**
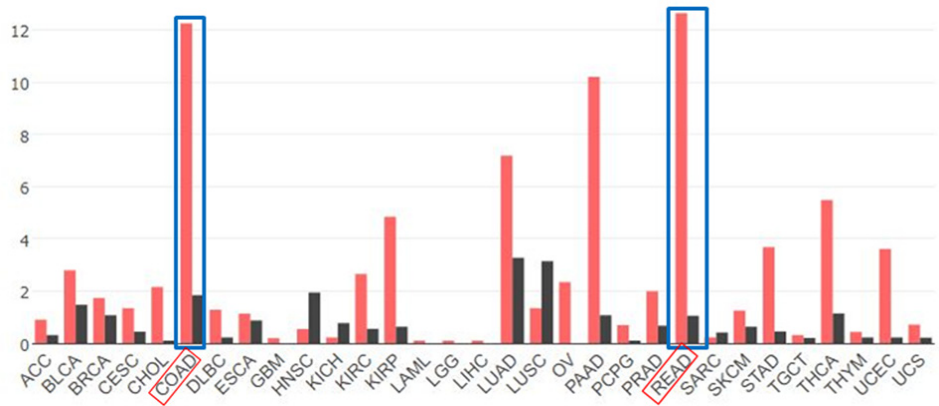
Screening out ABHD11-AS1 (WBSCR26) from the Cancer Genome Atlas (TCGA) in cancers by an interactive web server ‘GEPIA’ RNA-Seq data from TCGA of lncRNA ABHD11-AS1 in different cancers were analyzed, involved in colon cancer tissues (*n* = 275), paired normal colon tissue (*n* = 349) and rectal cancer tissues (*n* = 92), and paired normal rectal tissue (*n* = 316).

**Figure 2:**
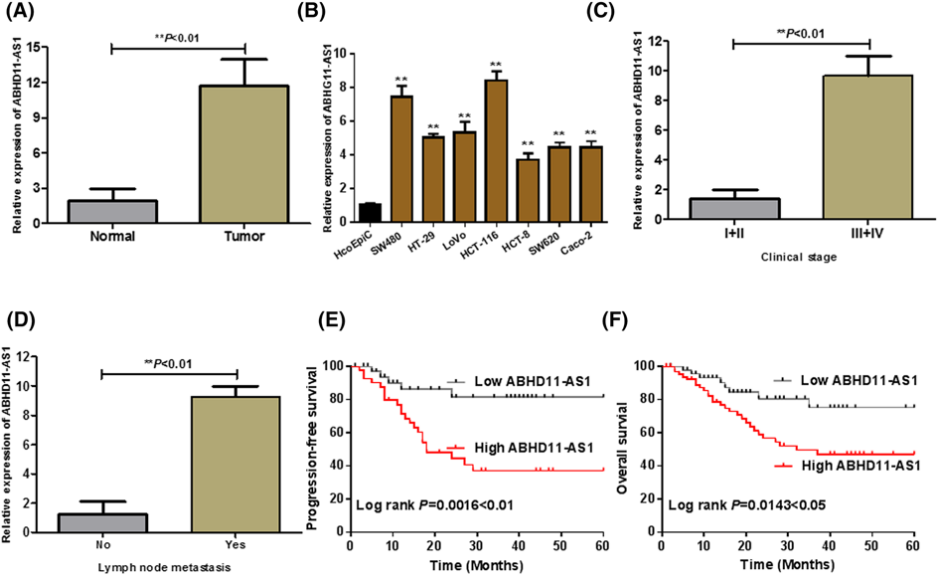
LncRNA ABHD11-AS1 expression was up-regulated in CRC and associated with poor prognosis of CRC patients (**A**) The expression of ABHD11-AS1 in CRC tissues (Tumor = **132**) was significantly higher than the pair-matched normal adjacent colorectal tissues (Normal = **132**) (**P < 0.01). (**B**) The expression of ABHD11-AS1 was down-regulated in a series of CRC cell lines compared with human colonic epithelial cell (HcoEpiC), **P < 0.01 compared with HcoEpiC. (**C**) The expression of ABHD11-AS1 in tumor tissues with high clinical stage (III–IV) was significantly higher than that of low clinical stage (I–II) (**P < 0.01). (**D**) The expression of ABHD11-AS1 in tumor tissues with lymph node metastasis was significantly higher than that without lymph node metastasis (**P < 0.01). (**E**) and (**F**) Higher expression of ABHD11-AS1 was associated with shorter the PFS or OS by Kaplan–Meier analysis and log rank test. *P < 0.05, **P < 0.01.

To explore the expression of ABHD11-AS1 in CRC, we analyzed RNA sequencing expression data of ABHD11-AS1 from the TCGA and the GTEx projects by an interactive web server ‘GEPIA’ (http://gepia.cancer-pku.cn/). As shown in Figure 1, the results clarified that lncRNA ABHD11-AS1 was the most overexpressed in colon and rectal cancer tissues compared with adjacent normal tissues among different kinds of cancer. Consistently, as shown in Figure 2A, qRT-PCR assay indicated that ABHD11-AS1 expression was also significantly up-regulated in CRC tissues (*n* = 132) from our hospital, compared with adjacent normal tissues (*n* = 132) (Figure 2A, *P* < 0.01). To further support the above discovery, we examined ABHD11-AS1 expression in seven CRC cell lines (SW480, HT-29, LoVo, HCT-116, HCT-8, SW620, Caco-2) and normal human colonic epithelial cell (HcoEpiC) by qRT-PCR. Similarly, as shown in Figure 2B, ABHD11-AS1 expression was also strikingly enhanced in CRC cell lines (*P* < 0.01). To better understand clinical significance of ABHD11-AS1, as shown in Figure 2C,D, we found that high level of ABHD11-AS1 was statistically associated with cTNM stage (**P < 0.01) and lymph node metastasis (**P < 0.01). In addition, we divided these CRC patients into two group according to the median expression of ABHD11-AS1: the high ABHD11-AS1 expression group (*n* = 66, fold-change ≥ median) and low ABHD11-AS1 expression group (*n* = 66, fold-change ≤ median). Kaplan–Meier analysis suggested that high ABHD11-AS1 expression significantly reduced PFS (Figure 2E, *P* = 0.0016 < 0.01) and OS (Figure 2F, *P* = 0.0143 < 0.05) of CRC patients.

### Knockdown of ABHD11-AS1 abrogated cell proliferation, colony formation, migration, and invasion, promoted cell apoptosis in CRC cells *in vitro*

**Figure 3:**
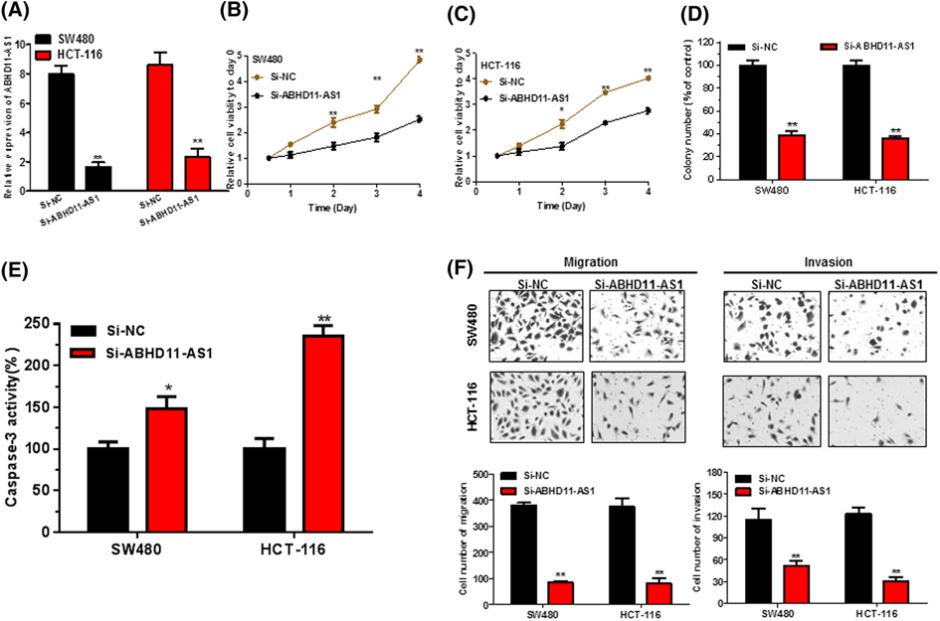
Knockdown of ABHD11-AS1 abrogated cell proliferation, colony formation, migration, and invasion, promoted cell apoptosis in CRC cells (**A**) ABHD11-AS1 expression levels were examined by qRT-PCR in siRNA transfected CRC cells. **P < 0.01 compared with Si-NC. (**B**) and (**C**) Knockdown of ABHD11-AS1 significantly inhibited cell proliferation of CRC cells by performing CellTiter-Glo assay. (**D**) Colony formation assay showed that knockdown of ABHD11-AS1 significantly reduced the colony-forming ability of CRC cells. (**E**) Knockdown of ABHD11-AS1 significantly promoted cell apoptosis by caspase-3 ELISA assay kit. (**F**) Knockdown of ABHD11-AS1 significantly inhibited migration and invasion of CRC cells by using transwell assay. Data were expressed as mean ± SD from three independent assay**.** * *P* <0.05, **P < 0.01.

SW480 and HCT-116 cells were used to investigate the effect of ABHD11-AS1 on biological function due to the higher expression of ABHD11-AS1 in the panel of CRC lines (Figure 2B). qRT-PCR assay suggested that ABHD11-AS1 was successfully knockdown by siRNA (Figure 3A, *P* < 0.01). Then, CellTiter-Glo assay elucidated that the proliferation capability of SW480 and HCT-116 cells was prominently inhibited (Figure 3B,C, *P* < 0.05). Subsequently, colony formation assay also indicated that knockdown of ABHD11-AS1 could significantly suppressed colony numbers of CRC cells (Figure 3D, *P* < 0.01). Besides, Caspase 3 ELISA assay confirmed that ABHD11-AS1 knockdown obviously promoted cell apoptosis (Figure 3E, *P* < 0.01). Next, transwell assay suggested that the migration and invasion abilities of CRC cells were remarkably reduced after ABHD11-AS1 knockdown (Figure 3F, *P* < 0.01).

### ABHD11-AS1 functioned as a sponge for miR-133a in CRC cell

**Figure 4:**
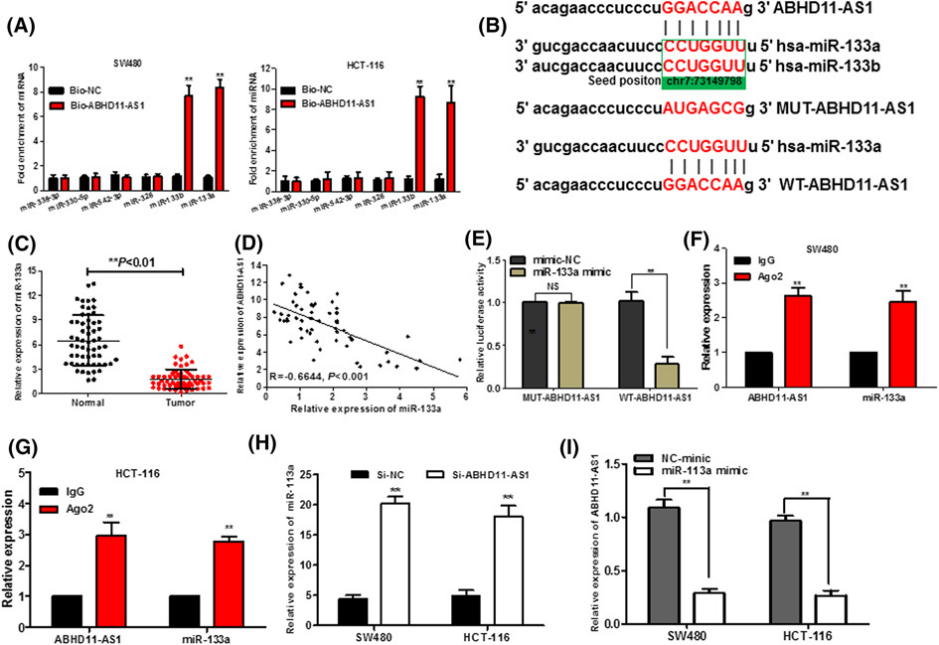
ABHD11-AS1 directly interacts with miR-133a (**A**) Expression of miRNAs was determined by using qRT-PCR in the sample pulled down by biotinylated ABHD11-AS1 probe. (**B**) Predict binding sites between ABHD11-AS1 and miR-133a/b. (**C**) The relative expression of miR-133a in tumor tissues was significantly down-regulated compared with that in adjacent normal tissues. (**D**) Pearson correlation analysis demonstrated that there was a remarkable negative correlation between miR-133a and ABHD11-AS1 expression in CRC tissues. (**E**) The relative luciferase activities were inhibited in the HEK-293 cells transfected with the reporter vector WT-ABHD11-AS1, not Mut-ABHD11-AS1. (**F**) and **(G**) The RNA immunoprecipitation assay was used to confirm that ABHD11-AS1 and miR-133a could directly bind to AGO2 in CRC cells. (**H**) Relative expression of miR-133a was determined by qRT-PCR in CRC cells transfected with si-ABHD11-AS1. (**I**) Relative expression of ABHD11-AS1 in CRC cells transfected with miR-133a mimics was examined by qRT-PCR. Data were expressed as mean ± SD from three independent experiments. *P < 0.05, **P < 0.01.

To explore whether ABHD11-AS1 functions by the means of sponging miRNA or acting as ceRNAs, bioinformatics tools (microRNA.org and miRcode) were used to dissect the potential microRNA binding sites of ABHD11-AS1. By using a database, we found out that six miRNAs (miR-133a, miR-133b, miR-338-3p, miR-330-5p, miR-542-3p, and miR-326), which all acted as tumor suppressors in CRC tumorigenesis, had a highly putative binding site with lncRNA ABHD11-AS1. Subsequently, we performed the biotin-labeled pull-down assay to further examine which miRNAs ABHD11-AS1 could directly interact with. We observed a obviously up-regulation of miR-133a/b in the SPRY4-IT1 pulled down pellet compared with control group as detected by qRT-PCR, but the amount of miR-338-3p, miR-330-5p, miR-542-3p, and miR-326 in the ABHD11-AS1 pulled down pellet had no remarkable alteration compared with control groups (Figure 4A). As miR-133a and miR-133b have the same seed sequences (Figure 4B) and they both play a suppressive functions in development of CRC [11,12,20,21], we chose miR-133a as the study target to analysis whether its expression was modulated by ABHD11-AS1. qPCR assay further verified that miR-133a expression was apparently reduced in the CRC tissues (Figure 4C), consistently with previous reports [11,12]. Intriguingly, Pearson’s correlation analysis confirmed that an inverse relationship was observed between ABHD11-AS1 and miR-133a expression levels in CRC tissues (Figure 4D, *R* = −0.6644, *P* < 0.001). To further investigate whether ABHD11-AS1 directly interacted with miR-133a, dual-luciferase reporter assay demonstrated that miR-133a mimic could significantly attenuate the luciferase activity of the WT-ABHD11-AS1, while not observed in that of MUT-ABHD11-AS1 (Figure 4D, *P* < 0.01). To elucidate whether RNA-induced silencing complex (RISC) might be implicated in the mutual repression between ABHD11-AS1 and miR-133a, RNA immunoprecipitation assay (RIP) were conducted with antibody against Ago2, a major component of RISC complex. As shown in Figure 4F,G, ABHD11-AS1 and miR-133a were significantly enriched in Ago2-pulled down pellet in SW480 and HCT-116 cells. Meantime, we found out that knockdown of ABHD11-AS1 significantly promoted the miR-133a expression (Figure 4H, *P* < 0.01), and the miR-133a mimic dramatically induced the inverse results (Figure 4I, *P* < 0.01), which suggested that there might be a reciprocal repression feedback loop between ABHD11-AS1 and miR-133a.

### SOX4 was a novel target of miR-133a in CRC cells

**Figure 5:**
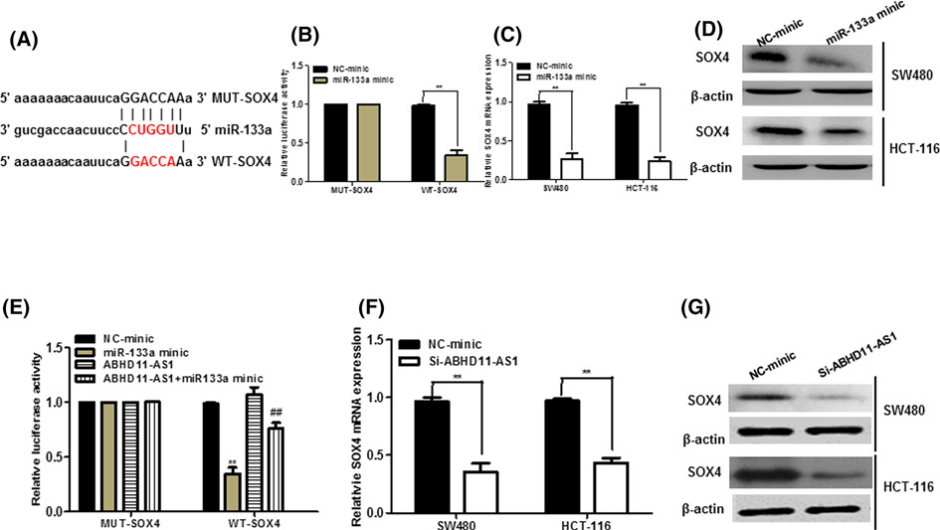
SOX4 was a novel target of miR-133a and positively regulated by ABHD11-AS1 (**A**) The 3′-UTR of SOX4 harbored miR-133a cognate site. (**B**) HEK-293 cells were co-transfected with miR-133a mimic and wild-type (WT) or mutant SOX4 3′-UTR for 48 h, the luciferase activity was measured. (**C**) and (**D**) SW480 and HCT-116 cells were transfected with miR-133a mimic for 48 h, the mRNA and protein level of SOX4 was measured. (**E**) HEK-293 cells were co-transfected with pcDNA-ABHD11-AS1 (ABHD11-AS1), miR-133a mimic and SOX4-5 3′-UTR for 48 h, the luciferase activity was measured. **P < 0.01, compared with NC-mimic. ##*P* < 0.01, compared with miR-133a mimic. (**F**) and (**G**) ABHD11-AS1 positively regulated the level of SOX4 mRNA and protein in SW480 and HCT-116 cells. **P < 0.01.

Bioinformatics analysis of TargetScan and miRBase predicted that miR-133a had the matched binding base with SOX4 (Figure 5A). Further, luciferase reporter assay showed that up-regulation of miR-133a significantly reduced SOX4 3′-UTR activity but had no effect on the mutant SOX4 3′-UTR activity (Figure 5B). Moreover, overexpression of miR-133a prominently abrogated the SOX4 mRNA and protein expression in CRC cells (Figure 5C,D). To explore whether ABHD11-AS1 competitively inhibited the binding of miR-133a to SOX4, we performed luciferase reporter assays. The results uncovered that ABHD11-AS1 could counteract the inhibitory effect of miR-141 on SOX4 (Figure 5E). In addition, silencing of ABHD11-AS1 significantly inhibited mRNA and protein level of SOX4 in CRC cells (Figure 5E,F).

### MiR-133a/SOX4 mediated the effect of ABHD11-AS1 on cell viability, apoptosis, and migration on CRC cell

**Figure 6:**
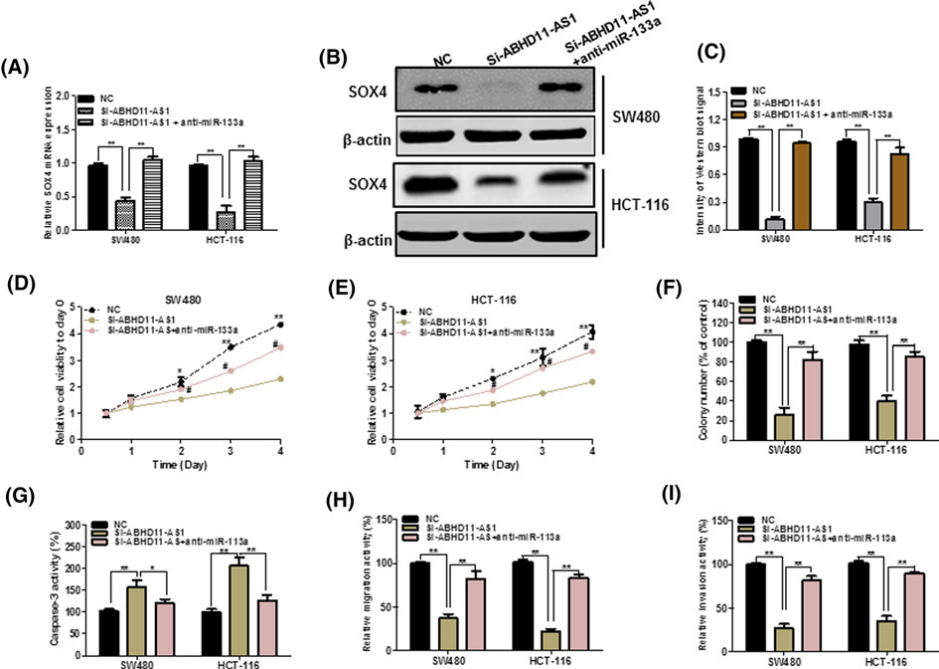
miR-133a mediated the tumorigenic effects of ABHD11-AS1 in CRC cells (**A**) and (**B**) miR-133a knockdown increased the mRNA and protein expression of SOX4 in ABHD11-AS1 knockdown. (**C**) Quantify the signal intensity of Western blots. (**D**) and (**E**) CellTiter-Glo assays showed that miR-133a could largely reverse the suppressive effects of ABHD11-AS1 knockdown on proliferation. (**F**) miR-133a silencing partly rescued the colony formation of CRC cells after ABHD11-AS1 knockdown. (**G**) Down-regulation of miR-133a largely decreased the promotion of apoptosis in CRC cells transfected with si-ABHD11-AS1. (**H**) and (**I**) miR-133a silencing partly reversed the suppressive effects of ABHD11-AS1 on migration and invasion. *P < 0.05, **P < 0.01.

Next, we tried to figure out whether ABHD11-AS1 could exert its biological functions through ABHD11-AS1/miR-133a/SOX4 axis. As illustrated in Figure 5E, up-regulation of ABHD11-AS1 significantly abolished the inhibitory effects of miR-133a overexpression on SOX4 3′-UTR activity. Meantime, as shown in Figure 6A–C, miR-133a knockdown inhibited the inhibitory effects of ABHD11-AS1 silencing on SOX4 expression in CRC cells. Functionally, celltiter-glo assay and colony formation assay exhibited that miR-133a inhibitor could alter the prohibited effect of ABHD11-AS1 down-regulation on growth of CRC cells (Figure 6D–F, *P* < 0.05). Besides, as seen from Figure 6G, miR-133a knockdown could abolish the promotion of apoptotic cells upon ABHD11-AS1 down-regulation. Transwell assay also demonstrated that miR-133a knockdown could reduce inhibitory effect of migration and invasion in CRC cells transfected with si-ABHD11-AS1 (Figure 6H,I).

## Discussion

Accumulating evidence revealed that lncRNAs play important roles in the tumorigenesis of CRC [22,23]. For instance, lncRNA-FEZF1-AS1 promotes tumor proliferation and metastasis in CRC by regulating PKM2 signaling [24]. SNHG5 promotes CRC cell survival by counteracting STAU1-mediated mRNA destabilization [25]. LncRNA ABHD11 Antisense RNA 1 (ABHD11-AS1) has been identified to act as diagnostic and prognostic bio-markers as well as therapeutic target in endometrial carcinoma, ovarian cancer, bladder cancer, gastric cancer [7–10]. However, the roles and underlying mechanisms of ABHD11-AS1 was unknown in CRC.

In our study, we for the first time showed that lncRNA ABHD11-AS1 was significantly overexpressed, and ABHD11-AS1 expression could be one prognostic marker of CRC patients. As revealed by functional experiments, ABHD11-AS1 knockdown inhibited biological function of CRC cell. These results fully indicated that ABHD11-AS1 acted an oncogene in the development of CRC, as consistent with previous studies that ABHD11-AS1 exerted oncogenic role in other tumors [7–10].

Numerous studies have found that lncRNAs act as ceRNAs for miRNAs to regulate tumor development and progression. Initially, bioinformatics analysis and RNA pull-down assay showed that biotin-labeled ABHD11-AS1 significantly pull-down miR-133a. Interestingly, we observed an inverse correlation between ABHD11-AS1 and miR-133a. Then, luciferase reporter assays further demonstrated that ABHD11-AS1 shared the miR-133a binding site. Most importantly, we found an endogenous interaction between ABHD11-AS1 and miR-133a by utilizing RIP with anti-Ago2 antibody. And a reciprocal repression of ABHD11-AS1 and miR-133a was also existed in CRC cells. Meanwhile, it was reported that miR-133a represses tumor growth and metastasis in CRC by targeting LIM and SH3 protein 1 and inhibiting the MAPK pathway [12] and miR-133a suppresses CRC cell invasion by targeting Fascin1 [26], indicating that miR-133a exerts tumor suppressor functions in CRC. So, ABHD11-AS1 exerted oncogenic effect on human CRC by negatively regulating miR-133a expression.

Then bioinformatics tools were used to predict the potential downstream targets of miR-133a. The analysis displayed that SOX4 might be a downstream target of miR-133a. In previous studies, SOX4 has been confirmed to be an oncogene in CRC [15–17]. Besides, SOX4 had been confirmed as a target of miR-133a in esophageal cancer cells by targeting the EMT to suppress the migration and invasion [13]. Subsequently, we found that ABHD11-AS1 could positively regulate the expression of SOX4 via targeting miR-133a in CRC cells. Moreover, miR-133a mediated the tumor promoting role of ABHD11-AS1 in CRC cells. In all, these results demonstrated that ABHD11-AS1 could promote growth and migration via ABHD11-AS1/miR-133a/SOX4 axis in CRC.

In conclusion, our results firstly demonstrated that ABHD11-AS1 was up-regulated and related with poor prognosis of CRC patients. And ABHD11-AS1 may function as a ceRNA to increase SOX4 expression by sponging miR-133a, which consequently contributed to CRC growth and migration. These results indicated that ABHD11-AS1 could be a potential therapeutic target for CRC treatment.

## Abbreviations

ABHD11-AS1: ABHD11 antisense RNA 1
CRC: colorectal cancer
lncRNAs: long noncoding RNAs
OS: overall survival
PFS: progression-free survival
qRT-PCR: quantitative real-time polymerase chain reaction
WT: wild type
